# Extending the NIF DISCO framework to automate complex workflow: coordinating the harvest and integration of data from diverse neuroscience information resources

**DOI:** 10.3389/fninf.2014.00058

**Published:** 2014-05-28

**Authors:** Luis N. Marenco, Rixin Wang, Anita E. Bandrowski, Jeffrey S. Grethe, Gordon M. Shepherd, Perry L. Miller

**Affiliations:** ^1^Center for Medical Informatics, Yale University School of MedicineNew Haven, CT, USA; ^2^VA Connecticut Healthcare System, US Department of Veterans AffairsWest Haven, CT, USA; ^3^Department of Neurobiology, Yale University School of MedicineNew Haven, CT, USA; ^4^Department of Neurosciences, Center for Research in Biological Systems, University of California at San DiegoLa Jolla, CA, USA; ^5^Department of Anesthesiology, Yale University School of MedicineNew Haven, CT, USA; ^6^Department of Molecular, Cellular and Developmental Biology, Yale UniversityNew Haven, CT, USA

**Keywords:** data integration, database federation, database interoperation, neuroinformatics, biomedical informatics

## Abstract

This paper describes how DISCO, the data aggregator that supports the Neuroscience Information Framework (NIF), has been extended to play a central role in automating the complex workflow required to support and coordinate the NIF’s data integration capabilities. The NIF is an NIH Neuroscience Blueprint initiative designed to help researchers access the wealth of data related to the neurosciences available via the Internet. A central component is the NIF Federation, a searchable database that currently contains data from 231 data and information resources regularly harvested, updated, and warehoused in the DISCO system. In the past several years, DISCO has greatly extended its functionality and has evolved to play a central role in automating the complex, ongoing process of harvesting, validating, integrating, and displaying neuroscience data from a growing set of participating resources. This paper provides an overview of DISCO’s current capabilities and discusses a number of the challenges and future directions related to the process of coordinating the integration of neuroscience data within the NIF Federation.

## INTRODUCTION

Experimental and computational data in neuroscience increasingly overwhelms our ability to integrate it to give insight into the molecular and cellular basis of normal and diseased neuronal function. The problem is extreme in neuroscience because the data comes from a wide variety of disciplines. Tools are therefore urgently needed for automating the discovery, extraction, and organization of this data. This paper describes the current status of the DISCO framework that has been extended to play a central role in automating the complex workflow required to support and coordinate the data integration capabilities of the Neuroscience Information Framework (NIF). The NIF^1^ is an NIH Neuroscience Blueprint initiative designed to help researchers access the wealth of data related to the neurosciences available via the Internet (Gardner et al., 2008; Gupta et al., 2008; Bandrowski et al., 2012; Cachat et al., 2012). A central component is the NIF Federation, a searchable database that currently (as of January, 2014) contains data that is downloaded on an ongoing basis from over 231 data and information resources (for an updated list see, http://disco.neuinfo.org).

A user querying the NIF Federation typically receives results from a range of resources containing data relevant to the query. For most resources, further information about a particular data item can be obtained by linking directly to data stored within the resource itself.

The NIF Federation is growing as new resources are added, and as new data are downloaded from participating resources. A major challenge involves the need to keep the data contained within the NIF Federation up-to-date, since most of its information resources are accumulating new data on a regular basis that need to be downloaded to the NIF. In addition, data previously downloaded from a resource may need to be changed to reflect changes made to the data within the resource. Furthermore, the internal structure of a resource may periodically change, requiring that the logic that “harvests” data from that resource be modified.

DISCO (DISCOvery) was initially developed as a set of tools to assist in focused aspects of the process described above (Marenco et al., 2010). During the past several years the role of DISCO has expanded dramatically to play a central role in automating the complex data-pipeline workflow required. Examples of DISCO’s capabilities include the following.

• creating a new data res up a schedule for downloading new data from a resource,ource in the NIF Federation describing what data to extract and how to extract that data,
• setting up a schedule for downloading new data from a resource
• downloading the current data from a resource and comparing it to the previous version of that data if one exists,
• creating a new version of the data for a resource and putting it in a temporary (“beta”) file to allow it to be inspected and approved before it is officially loaded into the operational version of the NIF Federation,
• allowing the NIF staff to create views of the NIF Federation data with the help of a concept mapper, including integrated views that combine data from multiple resources,
• alerting the NIF staff if problems are detected in any of these activities, and helping coordinate the resolution of each problem,
• maintaining a record of all these activities as they occur.

This paper provides an overview of DISCO’s current capabilities and discusses some of the issues and challenges involved in coordinating the integration of neuroscience data within the NIF Federation.

## BACKGROUND

DISCO can be described as an extensible data aggregator designed to facilitate automated information integration from disparate data sources over time. To help accomplish this goal DISCO includes the following features: persistence of provenance storage representation, historical data tracking, semantic data mappings, and near real-time federated data synchronization.

The most commonly known aggregators are Web crawlers. These scan the content of Web pages on a regular basis and index the terms extracted from free text retrieved. Any data that is stored inside Web-accessible databases, however, is not scanned and is therefore “invisible” to Web crawlers. DISCO differs in that it uses resource-specific tailored logic to guide focused data extraction from a variety of Web-based data-presentation formats, including Internet-accessible databases.

Two general approaches that have been widely described for integrating data from multiple distributed databases are (1) a data warehouse approach and (2) a data federation approach. In a data warehouse, data from participating resources are downloaded to a central database where they can be queried locally in an integrated fashion. Examples of data warehouses in the life sciences and clinical medicine includes DWARF (Fischer et al., 2006) and i2b2 clinical data warehouse (Majeed and Röhrig, 2012). By contrast, in a data federation, the data is not downloaded to a central database but remains stored within each participating resource. The federation (1) allows the user to submit a query, (2) breaks that query down into a set of individual subqueries that are submitted to each appropriate resource, and (3) integrates the results returned. Examples of biomedical data federated systems include InterPro BioMart (Jones et al., 2011) and caGrid (Saltz et al., 2006).

The NIF Federation implements a hybrid approach. A central component of the NIF Federation is a searchable data warehouse that contains selected data elements from participating resources. A major advantage of this approach is that queries can be executed much faster since all the data is stored in one place. There are no network communication latencies and no issues of participating resources being temporarily unavailable. In addition, complex database joins can be particularly difficult to implement within a pure data federation when the results of intermediate database operations need to be transmitted over the network. The NIF Federation is a hybrid because in addition to the data stored in its data warehouse, a large amount of additional data stored in individual resources can be linked to from data elements stored in the warehouse. This allows the user to “drill down” to very detailed data (for example to raw data such as complex experimental results) once the user determines based on a search of the warehouse that this additional data is of potential interest.

Many data warehouses store the data they retrieve from participating databases in a normalized form, so that the combined data can be queried as one single source. The NIF Federation does not do this. Normalizing data from highly dynamic heterogeneous federation of resources would be prohibitively time-consuming, if not impossible. As a result, the NIF stores the data retrieved from each participating resource in separate resource-specific tables.

DISCO stores the data that it extracts from all resources in relational form. Each resource has its data stored in its own set of DISCO tables. This approach to data extraction and storage forms the foundation for DISCO that facilitates data integration, searching over multiple resources, and tracking over time. Integrated querying of content within the NIF Federation is supported by first examining the text content and annotation of the imported data and mapping these to NIFSTD, the NIF ontology (Bug et al., 2008; Imam et al., 2012). Queries can then be expressed using NIFSTD. In addition, the NIF Federation has established some integrated views of data from multiple resources that allow those data to be more tightly linked for query purposes.

## OVERVIEW OF THE DISCO FUNCTIONALITY

This section first provides an overview of DISCO’s capabilities, after which we describe DISCO’s operation in more detail using a number of Web screens that illustrate concretely various aspects of DISCO’s functionality. More detail about the function of DISCO as a whole can be found in the online DISCO User’s and Technical Manual^2^.

**FIGURE 1:**
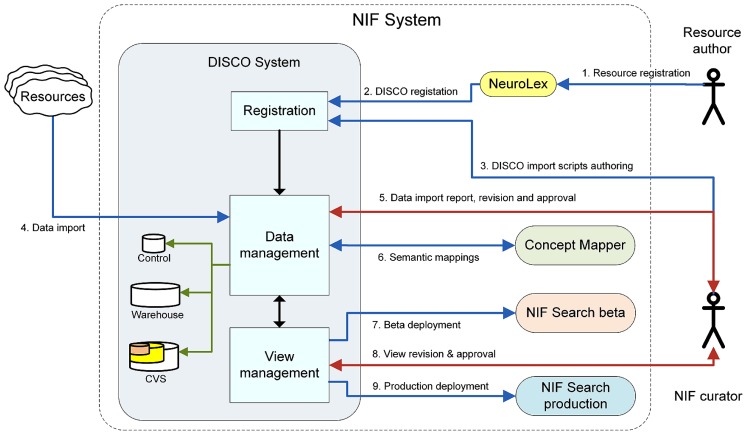
**A schematic overview of DISCO’s functionality**.

The current DISCO implementation represents a major advance over an early version of DISCO previously described in (Marenco et al., 2010). The previous version provided an initial set of tools that helped participating resource staff define the data they wished to include in the NIF Federation, and the upload of that data to the Federation. In its current implementation, DISCO provides a sophisticated infrastructure that coordinates and orchestrates three separate, but interrelated federated data processing pipelines for the NIF. The overall process coordinated by these pipelines is illustrated in **Figure 1**.

• The first pipeline involves DISCO resource registration. This process is initiated by creating a new resource sitemap at NeuroLex (neurolex.org). At the end of this initial step, this information is pushed to DISCO. Once registered in DISCO, NIF data curators then work with resource staff to construct DISCO scripts that extract data from the resource.
• The second pipeline involves input data management. Input data management includes ingestion of data from each resource, validation, and version tracking. These processes are entirely managed within the DISCO system.
• The third pipeline involves output data management. This process includes NIF Federation dataset view generation and validation, including deployment of that data for use by the NIF community. DISCO coordinates with other components of the NIF to generate federated data Views to help make the data accessible to users in a flexible fashion.

DISCO utilizes customized template scripts that describe to the NIF crawling agent where data is located for each resource, how it is to be extracted, and how it is to be stored in the NIF Federation warehouse. Source data in a variety of formats is supported, with new formats frequently incorporated. Within the warehouse, the resource data is stored using PostgreSQL tables. To facilitate provenance, tables from each resource are named using their NIF IDs as prefix. Every time a resource is rescanned, new temporary tables are generated. The new data is compared with the previous production version of the data for changes. If differences are found, the system reports a summary of the new changes to data curators for their verification. If the new data is accepted, DISCO checks whether there are any Views using this data. If so, new temporary Views are recreated and deployed to the NIF beta Website. Data curators as well as resource personnel are informed of a new version of these Views. Once the new temporary version of each View is approved, it is scheduled for production deployment. This process can be executed immediately or in batches, as desired.

DISCO was designed to deal with frequent changes in the information stored within a resource since such changes are common in neuroscience research. While most data changes consist of addition of new data or changes made to existing data, quite often a resource expands its content using new attributes or datasets. Less often the resource may reshape its contents using a different structure. Keeping track of these intra-resource domain changes over time is challenging unless these changes are properly documented within DISCO in a way that an automated agent can trace. DISCO scripts were particularly designed with this purpose in mind. Once a data extraction script is written, it is functional for data changes (additions, deletions, and corrections) as long as the structure of the data is not altered within the resource. If the structure changes, the extraction scripts need to be changed accordingly or the data ingestion procedure will break.

DISCO tracks data changes using predefined primary keys specified in resource scripts. (For a resource containing highly unstructured data, a hash of each entire record may be used.) These keys are used to create unique identifiers for specific pieces of data (entities) in a resource. The data structure within each resource is also tracked. DISCO uses a customized EAV/CR schema (Marenco et al., 2003) as a concurrent versioning system (CVS) back-end. Changes to data and metadata are stored using reverse delta methodology, and changes to resource database structures are stored using deltas. Reverse deltas allow DISCO to keep the most current version the data in the production tables actively used by the NIF, while changes from previous versions are stored in EAV form to allow the recreation of previous versions if needed. This technique is efficient for data additions and/or modest data edits. Substantial data changes may require copying all previous data to the CVS.

Semantic data mappings in DISCO are done based on schema annotations in DISCO scripts. We follow the approach to enhanced metadata annotation previously developed as the EAV/CR dataset protocol (EDSP; Marenco et al., 2003). Schema elements such as table groups, tables, and columns are annotated to describe their content, semantic relationships, and whether they contain complex objects, simple terms, or just values. Once data has been extracted from a resource, columns containing terms are queried to extract those terms, which are then mapped to term IDs from standard vocabularies. DISCO facilitates semantic data integration by mapping semantic metadata and term IDs to the NIFSTD ontology. The DISCO semantic data mapping functionality is coordinated by the NIF Concept Mapper (Gupta et al., 2008).

Having the most current data from each resource in DISCO is also challenging due to the absence of mechanisms to inform DISCO when new data is added to a resource. As described below, DISCO’s current scheduling approach allows the data to be refreshed at predefined intervals. There is therefore no assurance of having completely up-to-date data from each resource. We are currently exploring mechanisms to allow bidirectional notifications from resources to prompt DISCO when to rescan for new information. For resources that are not able to implement this mechanism, an approach using some type of probing may be possible, for example, checking for file timestamps or size changes.

Since DISCO is a system in continual evolution, for its development we use the Scrum agile development framework. This allows quick development and rollup into production. DISCO is implemented as a Web application written in Java using PostgreSQL as a back-end database.

## DISCO OPERATION

To coordinate its operation, DISCO contains three high-level “dashboards”: the main DISCO Dashboard, the NIF Data Sources Dashboard, and the NIF Views Dashboard. These are used by NIF staff to coordinate the various workflow steps required to maintain the NIF Federation. The three dashboards provide an overview of the status of all of DISCO’s various activities for every participating resource, along with the ability to drill down to see more detailed information about the activities and resources involved.

**FIGURE 2:**
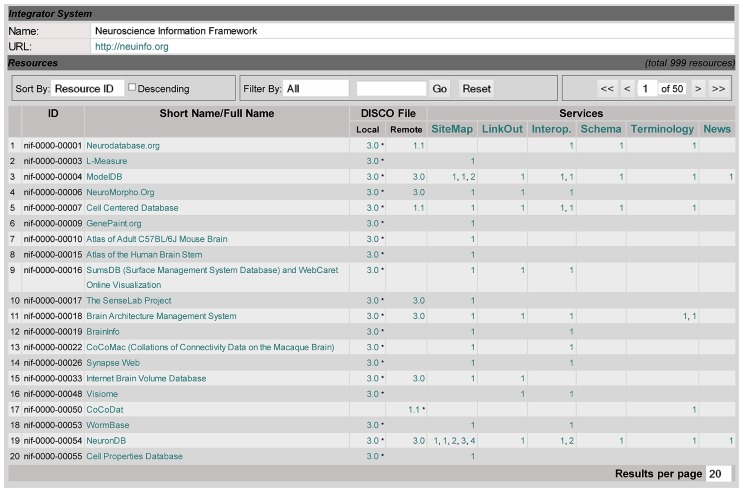
**The main DISCO Dashboard, as described in the text**.

**Figure 2** shows the main DISCO Dashboard, which also serves as DISCO’s homepage. It contains the list of the resources, including all the NIF Federation resources, that share their information via DISCO. This dashboard (at the top of the “Resource” section) shows a toolbar for searching, sorting, and paging through the resources. Below is a table of resources, showing each resource’s ID and name, as well as summary information indicating what NIF capabilities DISCO coordinates for that resource, as well as links to more detailed information. This information includes (1) where the DISCO file(s) for each resource resides (locally on the DISCO server or remotely at the resource itself), and (2) which DISCO services each resource is participating in. This dashboard provides NIF staff with an overview of this basic information about all participating resources.

**FIGURE 3:**
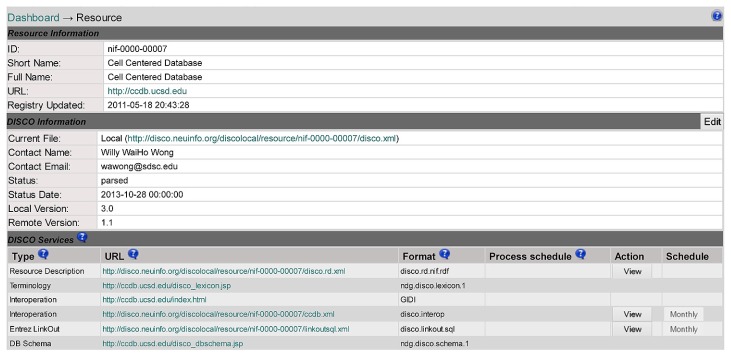
**A DISCO screen showing detailed information about an individual resource (Cell Centered Database) that participates in the NIF Federation**.

Clicking on a resource name (e.g., Cell Centered Database in line 5 of **Figure 2**) will link to the DISCO content page for that resource, as shown in **Figure 3**. This page displays a variety of information about that resource, including contact information for that resource’s technical support, as well as pointers to DISCO files which contain scripts defining in detail how DISCO implements each DISCO service for that resource (as summarized in the “Services” section of **Figure 2**). The content presented in this page is encoded using an XML formatted file, and can be modified by selecting the Edit button in the “DISCO Information” section of the page.

**FIGURE 4:**
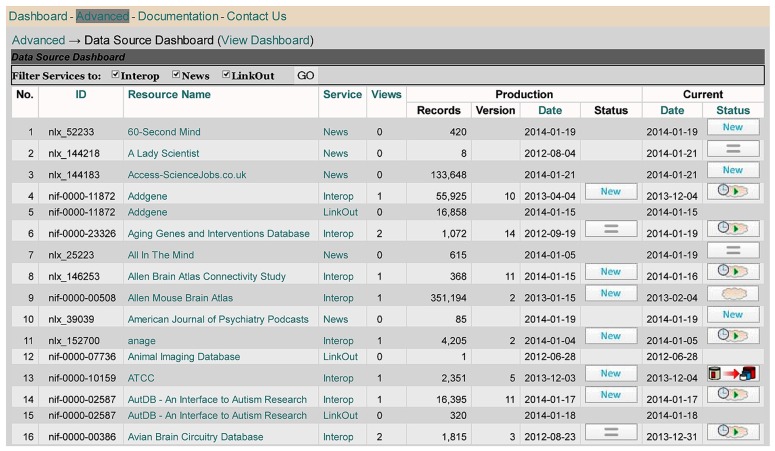
**DISCO’s NIF Data Source Dashboard**.

Whereas the main DISCO Dashboard provides an overview of the basic information DISCO maintains about each participating resource (including descriptive information and scripts), the NIF Data Source Dashboard (**Figure 4**) provides an overview of the *workflow status* of each resource.

**FIGURE 5:**
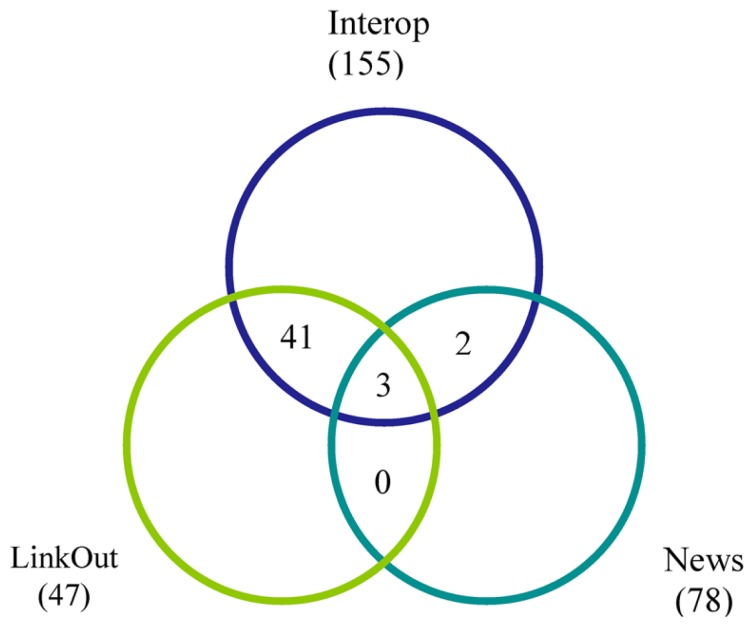
**A Venn diagram illustrating how the 231 resources supported by DISCO as of January 2014 are distributed among the three DISCO services (Interop, LinkOut, and News)**. The numbers indicate how many resources participate in each service and, of these, how many participate in two or three of the services.

The Data Source Dashboard displays a table with columns containing each resource’s ID and name, together with the DISCO service type provided and summary information concerning the “Production” and “Current” version of the data that has been uploaded to the NIF for each service. DISCO currently supports three types of service. The basic service (labeled Interop) involves incorporating a set of specified data from a resource into the NIF Federation’s data warehouse. The LinkOut service involves exporting data to the National Library of Medicine for incorporation in PubMed to support its ability to “link out” from a paper citation to related data items (Marenco et al., 2008). The News service allows DISCO to consolidate news provided by participating resources and to provide this aggregated news to interested NIF users. DISCO supports one or more of these three services for a total of 231 resources, as illustrated in **Figure 5**.

For example, as seen in **Figure 4**, the Addgene resource (lines 4 and 5) uses the DISCO Interop and LinkOut services. As indicated in **Figure 4**, the Addgene Interop data currently contains 55,925 records. The “Production” version of that data is in the 10th version of data uploaded, which was created on 4/4/13 and which involved the import of “New” data. In addition to this production version of the data, there is a more recent (“Current”) version of the data that was uploaded on 12/4/13, which is “Pending” (as indicated by the little clock icon) inspection and approval by NIF staff before it can be used as the production version. When the [ = ] icon is shown in the status column (as seen in line 2 of **Figure 4**), this indicates that the most recent version of the data downloaded was unchanged from the previous version.

Underlying the information presented on this screen is a formalized NIF Data Source Lifecycle, which includes the following workflow.

• NIF staffs specify how frequently the data for each resource should be updated. This is determined by NIF staff in consultation with resource staff and depends on how frequently new data is added to a resource.
• When the time comes to update the data, a new version of the data is uploaded into a temporary table, where it is held (marked as “pending”).
• The data is then compared against the production version of the data for that resource (unless of course this is the first version of data uploaded).
• If the data is unchanged from the production version, then that fact is recorded.
• If there is new data, and/or if previous data is changed, this fact is recorded, and the data continues to be held as “pending” until a NIF staff member reviews it (by inspecting the new data to assure that no errors or anomalies have occurred during the data import process).
• Based on this review, the NIF staff member may “approve” the newly uploaded version, in which case it will be scheduled for transfer to become the production version.
• If the NIF staff member identifies a potential problem, this fact is recorded. Depending on the nature of the problem a number of steps may take place next. Examples of the type of problems that occur when importing data include (1) data type errors, (2) duplicate keys, (3) text fields that are too big for the corresponding field within the NIF, and (4) failure of the data import process to complete.

This coordination of the data sources lifecycle is the heart of DISCO’s automated support of the workflow required to organize the ongoing harvest and integration of data from participating NIF Federation resources.

**FIGURE 6:**
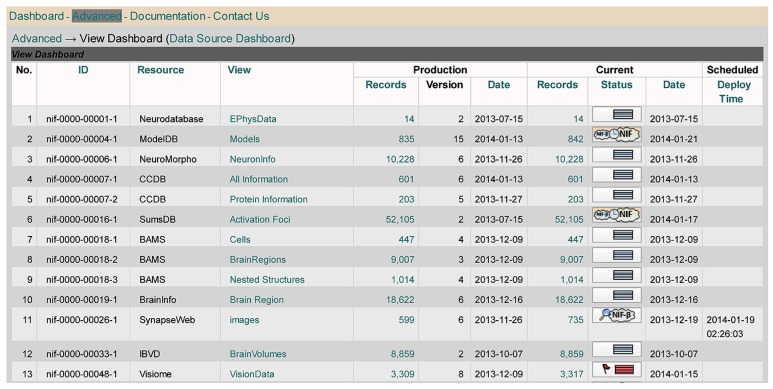
**DISCO’s NIF Views Dashboard**.

**Figure 6** shows NIF Views Dashboard, which coordinates the maintenance of the various views that have been defined over the NIF Federation data, including a growing number of views that combine data elements from multiple resources. Views involving multiple resources are “materialized” in the sense that data elements from the NIF tables for each of those resources are copied into new table. This allows the combined data to be queried and manipulated more efficiently. The decision to create such a view is made by NIF staff in consultation with members of a community of neuroscience researchers for whom such a view would be helpful in presenting data in a fashion that would be most intelligible. See the online DISCO Manual^3^ for more detail.

### SCHEDULING AND COORDINATING THE DATA UPDATE TASKS

**FIGURE 7:**
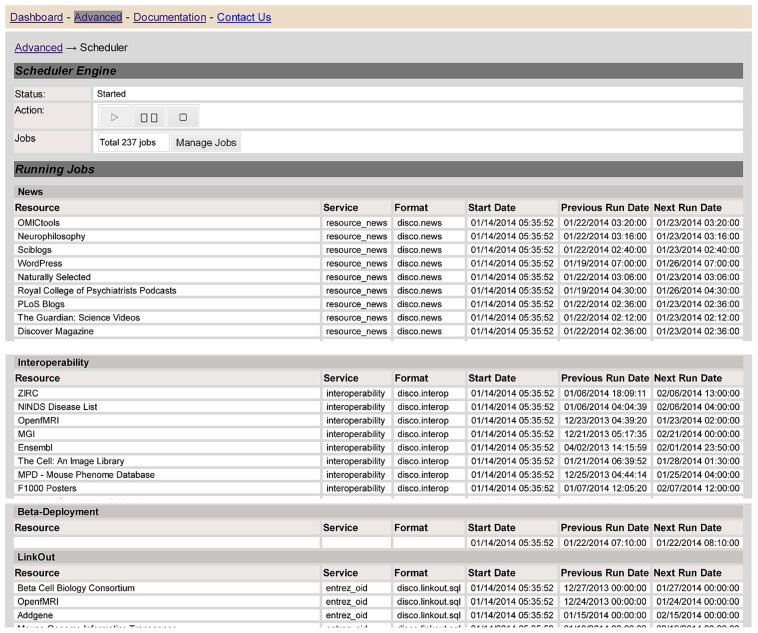
**The screen provides a snapshot overview that illustrates the operation of the DISCO task scheduler, as described in the text**.

At any given point of time, different resources will be at different points within the overall data life cycle, and there may be many tasks that are waiting to be executed or in the process of being executed. DISCO has a number of components to help manage, schedule and coordinate all these activities. To illustrate how DISCO manages these activities, **Figure 7** shows a Web page that provides an overview “snapshot” of the activities of the DISCO scheduler engine as of a given point of time. This table lists the various resources that are scheduled to be updated, or are in the process of being updated.

**FIGURE 8:**
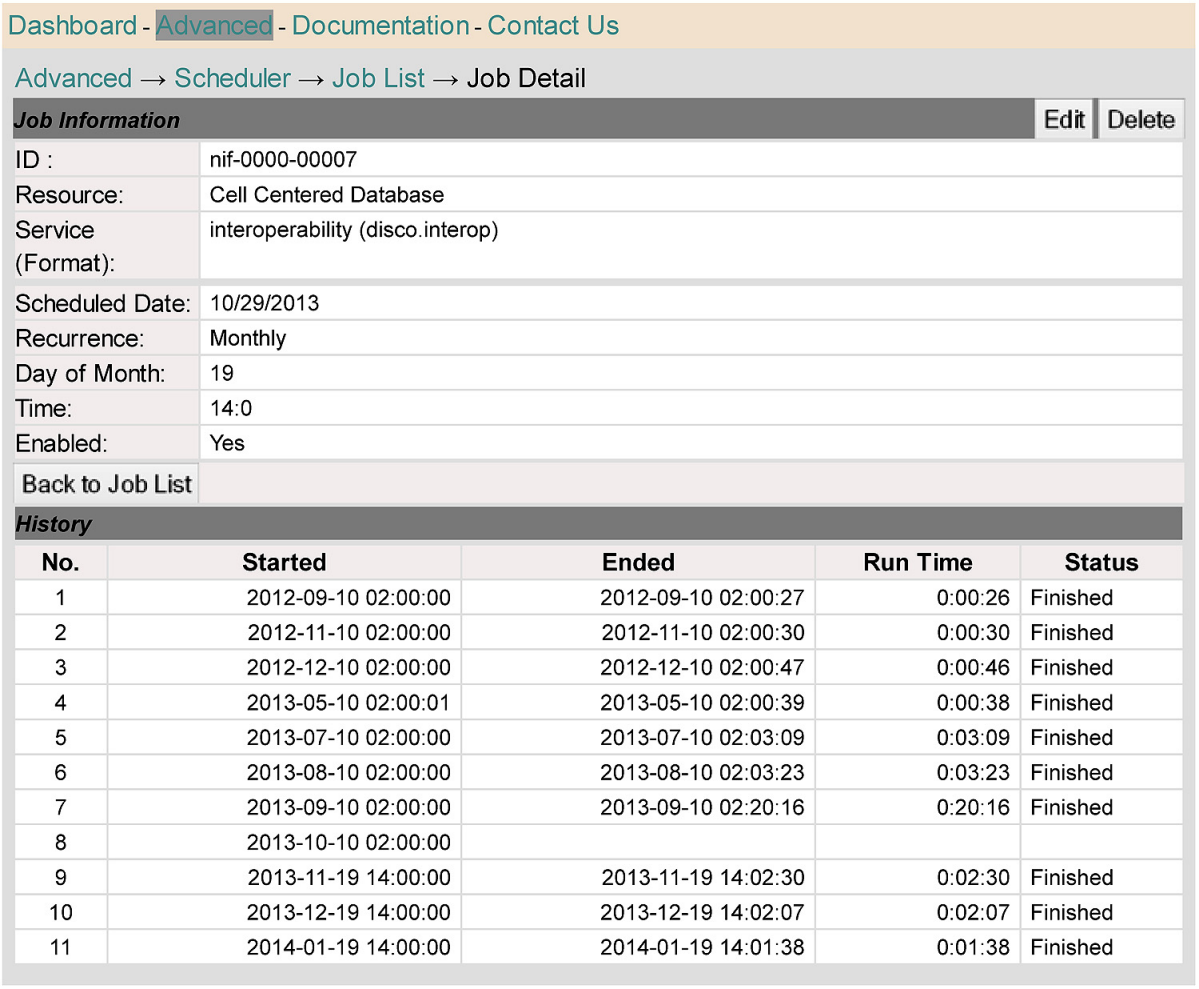
**This screen shows scheduling information relevant to a specific resource, the Cell Centered Database**.

**Figure 8** provides a different perspective on the scheduling function supported by DISCO. In this case, we see the process from the perspective of a single resource, in this case the Cell Centered Database. As indicated in the top half of the screen, the data for this resource is updated on a monthly basis, currently on the 19th of each month, at 2 P.M. The bottom half of the screen shows a record of the six most recent update runs.

This section has provided an overview of DISCO’s activities by showing a representative subset of the various screens that DISCO provides to help manage and coordinate the integration of data within the NIF Federation. Our goal in showing and describing these screens has been to help make the various functions that DISCO provides more concrete and transparent.

## CURRENT STATUS AND FUTURE DIRECTIONS

**FIGURE 9:**
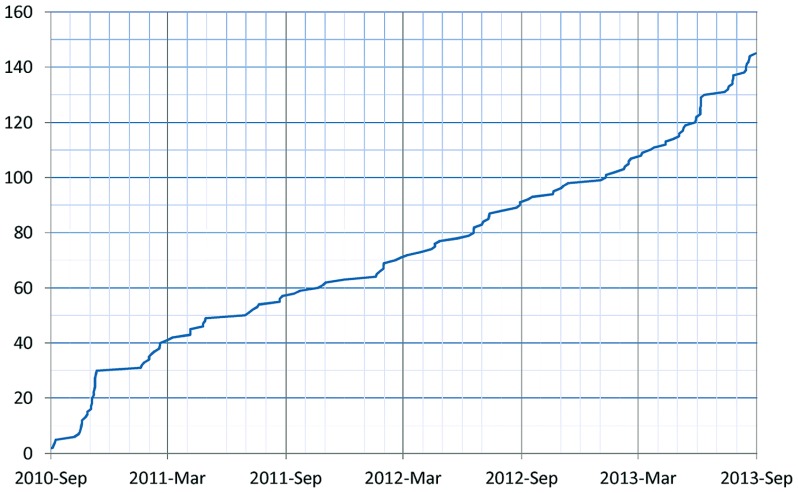
**Growth of the NIF Federation in terms of the number of participating resources over time**.

**FIGURE 10:**
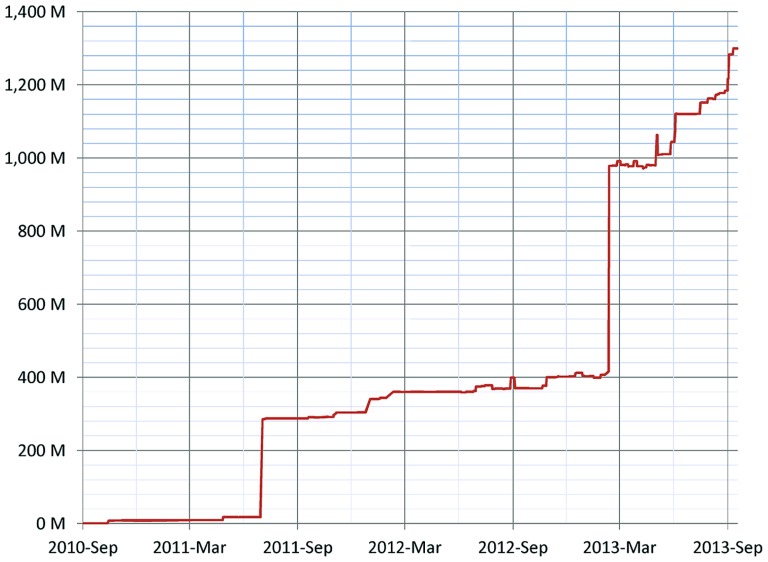
**Growth of the NIF Federation in terms of the number of records (the number of rows of data) stored over time**. There are two major jumps in seen in the graph: (1) in July 2011 BrainSpan.org (an atlas of the development of human brain) was added with 267 million records, and (2) in February 2013 PubMed was added with 567 million records.

**FIGURE 11:**
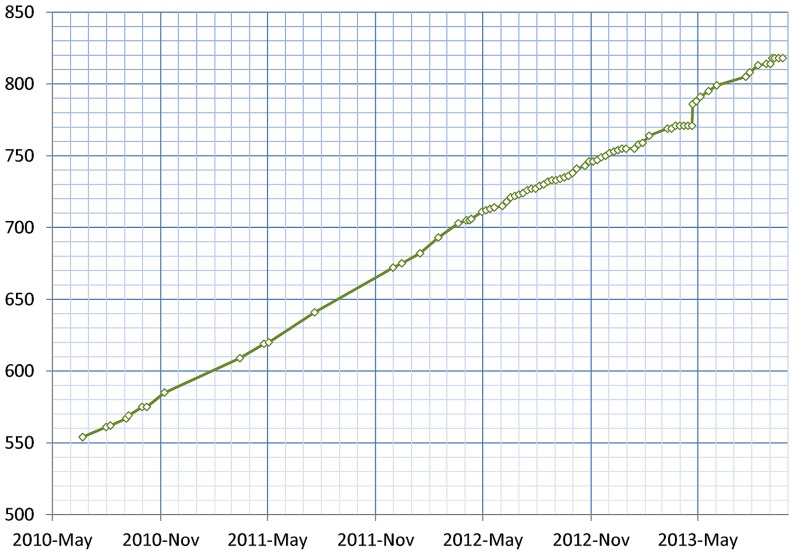
**This figures shows the growth of data from ModelDB stored in the NIF Federation, reflecting the growth of ModelDB itself, and the ongoing process of harvesting that data and integrating it into the NIF Federation**. Each diamond indicates a time when data was updated.

As of January 2014, 155 resources utilize the DISCO Interop service to share data via the NIF Federation. **Figure 9** shows how this number has gradually increased over time. The relatively steady rate of increase reflects the fact that the amount of effort to incorporate a new resource is relatively constant irrespective of the amount of data involved. **Figure 10** shows how the amount of data stored in the NIF Federation has increased over the same time period. **Table 1** indicates how frequently the NIF Federation resources are currently updated. **Figure 11** illustrates this process from the perspective of a single participating resource by showing how the amount of data stored in the NIF Federation for ModelDB has grown over time.

**Table 1:** This table shows how frequently the data from different Interop resources are updated within the NIF Federation, as of January 2014.

	# of resources
Weekly	12
Bi-weekly	4
Monthly	122
*Ad hoc* (not scheduled)	17
**Total**	155

Looking to the future, the development of DISCO will continue to be a work in progress. The overall approach is undergoing a continual process of refinement. There is also a quite extensive list of additional capabilities that would be desirable to incorporate in the future.

• There are a number of ways in which the current DISCO system could be refined and made more robust. For example, there is a need for more extensive status reporting and debugging tools for use when the import of data from a resource “hangs” (fails to complete). There are a wide variety of reasons this might happen, and it does happen quite regularly. It is a major problem that needs to be accommodated by providing as much automated assistance as possible.
• A second refinement that will be important as the NIF Federation grows will be to distribute DISCO’s functions over multiple machines so that the many tasks that are performed can be accomplished more rapidly utilizing parallel computing. DISCO currently runs on a single server machine.
• In addition to enhancing the current DISCO framework, there is a wide range of further capabilities that we would like to build. A major project would be to integrate the NIF’s ontology mapping functions so that they can be applied in an automated fashion to data as they are being imported. It is also becoming evident that the underlying capabilities implemented in DISCO could be utilized in other data aggregator systems, and that other groups would like to leverage the DISCO code. Some of these will want to extract data from some of the same resources as DISCO. The DISCO code could be adapted to facilitate its use by other groups including the shared harvesting of data from a resource by multiple aggregator systems.

## DISCUSSION

This section discusses some of the challenges that face the various groups of people who participate in developing and maintaining NIF and DISCO, including (1) the DISCO developers, (2) the NIF staff who use DISCO to coordinate their activities, and (3) the local staff at the participating resources. The biggest challenge facing all of these groups is that Web-based resources can be very idiosyncratic and difficult to extract data from, for a variety of reasons.

### LACK OF DESCRIPTIVE METADATA

One example of this problem is seen when a table containing data to be downloaded into the NIF does not include descriptive metadata such as informative column headers (e.g., a table might contain several idiosyncratic column names (“str1,”“str2,”…) without any metadata indicating that these columns contain the names of “strains”) or descriptive data types (e.g., a table might contain “1” as a data type instead of “male”). When descriptive, informative metadata is used in the tables downloaded from a resource, that metadata can in turn be used to more effectively annotate the data within the NIF to facilitate searching that data and integrating it with other data sets.

### USE OF COMPLEX DATA-PRESENTATION LOGIC

Another problem is seen when resource developers use customized client-side code (such as JavaScript) to present data in their local Web site. This approach has the advantage of allowing the Web presentation of the data to be “flashier” and potentially more understandable by the resource’s users. Unfortunately for DISCO staff, the approach also results in the data being in effect concealed by the client-side programming. To extract data from such a Web site, DISCO staff must examine and understand this code in detail to understand exactly what it is doing, so that they can write appropriate logic to access the data.

• A simple approach that greatly facilitates data extraction from a Web-based resource is for the resource developers to use a standardized template to organize the data presented on each Web page. For example, a standard set of section headers that are located consistently within each Web page greatly facilitates the organized extraction of that data. In addition, using standard terms for column headers greatly facilitates semi-automated terminology mapping of resource terms by the NIF Concept Mapper (Gupta et al., 2008).

It will be important to develop guidelines and standards for resource Web-site design that can be used by a resource to facilitate incorporation into a system like DISCO. New Web standards such as HTLM5, RDFa, and Google Microformats are very productive steps in this direction since they encourage and facilitate the incorporation of semantic metadata describing a resource’s data. The increasing use of these approaches in the design of Web resources will facilitate automated data extraction by data aggregators such as DISCO.

## INFORMATION SHARING STATEMENT

Technical information describing DISCO, including installation instructions, is available at: http://disco.neuinfo.org/docs/manual/. Additional program code can be obtained by contacting project staff.

## Conflict of Interest Statement

The authors declare that the research was conducted in the absence of any commercial or financial relationships that could be construed as a potential conflict of interest.

## Footnotes

1 http://www.neuinfo.org

2 http://disco.neuinfo.org/docs/manual/

3 http://disco.neuinfo.org/docs/manual/

## References

[B1] Bandrowski A. E. Cachat J. Li Y. Müller H. M. Sternberg P. W. Ciccarese P. (2012). A hybrid human and machine resource curation pipeline for the Neuroscience Information Framework. *Database (Oxford)* 2012:bas005 10.1093/database/bas005 PMC3308161 22434839

[B2] Bug W. J. Ascoli G. A. Grethe J. S. Gupta A. Fennema-Notestine C. Laird A. R. (2008). The NIFSTD and BIRNLex vocabularies: building comprehensive ontologies for neuroscience. *Neuroinformatics* 6 175–194 10.1007/s12021-008-9032-z 18975148 PMC2743139

[B3] Cachat J. Bandrowski A. Grethe J. S. Gupta A. Astakho V. Imam F. (2012). A survey of the neuroscience resource landscape: perspectives from the neuroscience information framework. *Int. Rev. Neurobiol.* 103 39–68 10.1016/B978-0-12-388408-4.00003-4 23195120

[B4] Fischer M. Thai Q. K. Grieb M. Pleiss J. (2006). DWARF – a data warehouse system for analyzing protein families. *BMC Bioinformatics* 7:495 10.1186/1471-2105-7-495 PMC1647292 17094801

[B5] Gardner D. Akil H. Ascoli G. A. Bowden D. M. Bug W. Donohue D. E. (2008). The Neuroscience Information Framework: a data and knowledge environment for neuroscience. *Neuroinformatics* 6 149–160 10.1007/s12021-008-9024-z 18946742 PMC2661130

[B6] Gupta A. Bug W. Marenco L. Qian X. Condit C. Rangarajan A. (2008). Federated access to heterogeneous information resources in the Neuroscience Information Framework (NIF). *Neuroinformatics* 6 205–217 10.1007/s12021-008-9033-y 18958629 PMC2689790

[B7] Imam F. T. Larson S. D. Bandrowski A. Grethe J. S. Gupta A. Martone M. E. (2012). Development and use of Ontologies Inside the Neuroscience Information Framework: a Practical Approach. *Front. Genet.* 3:111 10.3389/fgene.2012.00111 PMC3381282 22737162

[B8] Jones P. Binns D. McMenamin C. McAnulla C. Hunter S. (2011). The InterPro BioMart: federated query and web service access to the InterPro Resource. *Database (Oxford)* 2011:bar033 10.1093/database/bar033 PMC3170169 21785143

[B9] Majeed R. W Röhrig R. (2012). Automated realtime data import for the i2b2 clinical data warehouse: introducing the HL7 ETL cell. *Stud. Health Technol. Inform.* 180 270–274 22874194

[B10] Marenco L. Giorgio A. Ascoli G. A. Martone M. E. Shepherd G. M. Miller P. L. (2008). The NIF LinkOut Broker: a web resource to facilitate federated data integration using NCBI identifiers. *Neuroinformatics* 6 219–227 10.1007/s12021-008-9025-y 18975149 PMC2704600

[B11] Marenco L. Tosches N. Crasto C. Shepherd G. Miller P. L. Nadkarni P. M. (2003). Achieving evolvable Web-database bioscience applications using the EAV/CR framework: recent advances. *J. Am. Med. Inform. Assoc.* 10 444–453 10.1197/jamia.M1303 12807806 PMC212781

[B12] Marenco L. Wang R. Shepherd G. M. Miller P. L. (2010). The NIF DISCO Framework: facilitating automated integration of neuroscience content on the web. *Neuroinformatics* 8 101–112 10.1007/s12021-010-9068-8 20387131 PMC3819210

[B13] Saltz J. Oster S. Hastings S. Langella S. Kurc T. Sanchez W. (2006). caGrid: design and implementation of the core architecture of the cancer biomedical informatics grid. *Bioinformatics* 22 1910–1916 10.1093/bioinformatics/btl272 16766552

